# Supplementary material for: Epidemiology and survival outcomes of HIV-associated cervical cancer in Nigeria
Source: Infect Agent Cancer. 2023 Nov 1;18:68. doi: 10.1186/s13027-023-00550-7 (PMC10619301; doi:10.1186/s13027-023-00550-7)
Supplement: Supplementary file 1 — Additional file 1. Institutional Ethics Approvals. [file 13027_2023_550_MOESM1_ESM.pdf]

Northwestern | RESEARCH

Northwestern University
Institutional Review Board
Biomedical IRB
750 N. Lake Shore Dr., 7th Fl.
Chicago, Illinois 60611

irb@northwestern.edu
Office 312. 503. 9338

Social & Behavioral Sciences IRB
600 Foster St., 2nd Floor
Evanston, Illinois 60208

sbsirb@northwestern.edu
Office 847. 467. 1723

APPROVAL OF CONTINUING REVIEW

DATE: May 14, 2019

TO: Lifang Hou
FROM: Office of the IRB

DETERMINATION DATE: 5/14/2019
APPROVAL DATE: 5/13/2019
EXPIRATION DATE: 5/12/2020

The Northwestern University IRB reviewed and approved the submission described below:

Type of Submission:	Continuing Review
Review Level:	Committee
Expedited Category:	N/A
Title of Study:	Epigenetic biomarkers of cervical cancer in Nigerian Women
Principal Investigator:	Lifang Hou
IRB ID:	STU00207051-CR0001
Funding Source:	Name: National Cancer Institute, Grant Office ID: SP0041943, Funding Source ID: U54CA221205
Grant ID:	None
IND, IDE, or HDE:	None
Documents Reviewed:	• JUTH Ethics Review Approval_Cervical , Category: Approval/Authorization Letter;<br>• Consent Form , Category: Consent Form;<br>• Musa_CITI Certification , Category: Certificate;<br>• LUTH_RecruitmentFlyer, Category: Recruitment Materials;<br>• Anorlu_CITICertification, Category: Certificate;<br>• NU_eIRB_Cervical_Cancer project_2018_Feb9 revised 21Feb2018.docx, Category: IRB Protocol;<br>• Full Grant , Category: Sponsor Attachment;<br>• Sagay_CITI Certification , Category: Certificate;<br>• LUTH_Ethical Review Approval_Cervical , Category: Approval/Authorization Letter;<br>• JUTH_RecruitmentFlyer, Category: Recruitment Materials;<br>• Protocol , Category: IRB Protocol;<br>• Ogunsola_CITICertification , Category: Certificate;

In conducting this study, you are required to follow the requirements listed in the Northwestern University (NU) Investigator Manual (HRP-103), which can be found by navigating to the IRB Library within the eIRB+ system.

If you are unable to complete the study within the approval period, you will need to submit a continuing review to renew the study. The continuing review must be submitted no more than 60 days prior to the expiration date.

NU IRB approval does not constitute or guarantee institutional approval and/or support. Investigators and study team members must comply with all applicable federal, state, and local laws, as well as NU Policies and Procedures, which may include obtaining approval for your research activities from other individuals or entities.

For IRB-related questions, please consult the NU IRB website at http://irb.northwestern.edu. For general research questions, please consult the NU Office for Research website at www.research.northwestern.edu.

Northwestern | RESEARCH

Northwestern University
Institutional Review Board
Biomedical IRB
750 N. Lake Shore Dr., 7th Fl.
Chicago, Illinois 60611

irb@northwestern.edu
Office 312. 503. 9338

Social & Behavorial Sciences IRB
600 Foster St., 2nd Floor
Evanston, Illinois 60208

sbsirb@northwestern.edu
Office 847. 467. 1723

APPROVAL OF CONTINUING REVIEW

DATE: April 8, 2020

TO: Lifang Hou
FROM: Office of the IRB

DETERMINATION DATE: 4/6/2020
APPROVAL DATE: 4/6/2020
EXPIRATION DATE: 4/5/2021

The Northwestern University IRB reviewed and approved the submission described below:

Type of Submission:	Continuing Review
Review Level:	Committee
Expedited Category:	N/A
Title of Study:	Epigenetic biomarkers of cervical cancer in Nigerian Women
Principal Investigator:	Lifang Hou
IRB ID:	STU00207051-CR0002
Funding Source:	Name: National Cancer Institute, Grant Office ID: SP0041943, Funding Source ID: U54CA221205
Grant ID:	None
IND, IDE, or HDE:	None
Documents Reviewed:	• JUTH Ethics Review Approval_Cervical , Category: Approval/Authorization Letter;<br>• Consent Form , Category: Consent Form;<br>• Anorlu_CITICertification 2.0, Category: Certificate;<br>• JUTH Ethcial Clearance Approval_2.1, Category: Approval/Authorization Letter;<br>• Musa_CITI Certification , Category: Certificate;<br>• LUTH_RecruitmentFlyer, Category: Recruitment Materials;<br>• Anorlu_CITICertification, Category: Certificate;<br>• Sagay_CITI Certification , Category: Certificate;<br>• Sagay_CITI Certification 2.0, Category: Certificate;<br>• LUTH_Ethical Review Approval_Cervical , Category: Approval/Authorization Letter;<br>• JUTH_RecruitmentFlyer, Category: Recruitment Materials;<br>• LUTH_Ethical Review Approval_2.1, Category: Approval/Authorization Letter;<br>• Ogunsola_CITICertification , Category: Certificate;<br>• Revised Protocol , Category: IRB Protocol
Special Determinations:	none

In conducting this study, you are required to follow the requirements listed in the Northwestern University (NU) Investigator Manual (HRP-103), which can be found by navigating to the IRB Library within the eIRB+ system.

If you are unable to complete the study within the approval period, you will need to submit a continuing review to renew the study. The continuing review must be submitted no more than 60 days prior to the expiration date.

NU IRB approval does not constitute or guarantee institutional approval and/or support. Investigators and study team members must comply with all applicable federal, state, and local laws, as well as NU Policies and Procedures, which may include obtaining approval for your research activities from other individuals or entities.

For IRB-related questions, please consult the NU IRB website at http://irb.northwestern.edu. For general research questions, please consult the NU Office for Research website at www.research.northwestern.edu.

Northwestern | RESEARCH

Northwestern University
Institutional Review Board
Biomedical IRB
750 N. Lake Shore Dr., 7th Fl.
Chicago, Illinois 60611

irb@northwestern.edu
Office 312. 503. 9338

Social & Behavioral Sciences IRB
600 Foster St., 2nd Floor
Evanston, Illinois 60208

sbsirb@northwestern.edu
Office 847. 467. 1723

APPROVAL OF CONTINUING REVIEW

DATE: March 16, 2021

TO: Lifang Hou
FROM: Office of the IRB

DETERMINATION DATE: 3/16/2021
APPROVAL DATE: 3/15/2021
EXPIRATION DATE: 3/14/2022

The Northwestern University IRB reviewed and approved the submission described below:

Type of Submission:	Continuing Review
Review Level:	Committee
Expedited Category:	N/A
Title of Study:	Epigenetic biomarkers of cervical cancer in Nigerian Women
Principal Investigator:	Lifang Hou
IRB ID:	STU00207051-CR0003
Funding Source:	Name: National Cancer Institute, Grant Office ID: SP0041943, Funding Source ID: U54CA221205
Grant ID:	None
IND, IDE, or HDE:	None
Documents Reviewed:	• JUTH Ethics Review Approval_Cervical , Category: Approval/Authorization Letter;<br>• Consent Form , Category: Consent Form;<br>• Anorlu_CITICertification 2.0, Category: Certificate;<br>• JUTH Ethcial Clearance Approval_2.1, Category: Approval/Authorization Letter;<br>• Musa_CITI Certification , Category: Certificate;<br>• LUTH_RecruitmentFlyer, Category: Recruitment Materials;<br>• Anorlu_CITICertification, Category: Certificate;<br>• Sagay_CITI Certification , Category: Certificate;<br>• Sagay_CITI Certification 2.0, Category: Certificate;<br>• LUTH_Ethical Review Approval_Cervical , Category: Approval/Authorization Letter;<br>• JUTH_RecruitmentFlyer, Category: Recruitment Materials;<br>• LUTH_Ethical Review Approval_2.1, Category: Approval/Authorization Letter;<br>• Ogunsola_CITICertification , Category: Certificate;<br>• Revised Protocol , Category: IRB Protocol;<br>• LUTH IRB Approval, Category: Continuing Review Supporting

	Document;
Special Determinations:	None
Unaffiliated External Site(s) that rely on NU IRB:	• Lagos University Teaching Hospital<br>• Jos University Teaching Hospital
Clinical Trial:	No

In conducting this study, you are required to follow the requirements listed in the Northwestern University (NU) Investigator Manual (HRP-103), which can be found by navigating to the IRB Library within the eIRB+ system.

If you are unable to complete the study within the approval period, you will need to submit a continuing review to renew the study. The continuing review must be submitted no more than 60 days prior to the expiration date.

NU IRB approval does not constitute or guarantee institutional approval and/or support. Investigators and study team members must comply with all applicable federal, state, and local laws, as well as NU Policies and Procedures, which may include obtaining approval for your research activities from other individuals or entities.

For IRB-related questions, please consult the NU IRB website at http://irb.northwestern.edu. For general research questions, please consult the NU Office for Research website at www.research.northwestern.edu.

Northwestern | RESEARCH

Northwestern University
Institutional Review Board
Biomedical IRB
750 N. Lake Shore Dr., 7th Fl.
Chicago, Illinois 60611

irb@northwestern.edu
Office 312. 503. 9338

Social & Behavorial Sciences IRB
600 Foster St., 2nd Floor
Evanston, Illinois 60208

sbsirb@northwestern.edu
Office 847. 467. 1723

APPROVAL OF CONTINUING REVIEW

DATE: April 5, 2022

TO: Lifang Hou
FROM: Office of the IRB

DETERMINATION DATE: 4/4/2022
APPROVAL DATE: 4/4/2022
EXPIRATION DATE: 4/3/2023

The Northwestern University IRB reviewed and approved the submission described below:

Type of Submission:	Continuing Review
Review Level:	Committee
Title of Study:	Epigenetic biomarkers of cervical cancer in Nigerian Women
Principal Investigator:	Lifang Hou
IRB ID:	STU00207051-CR0004
Funding Source:	Name: National Cancer Institute, Grant Office ID: SP0041943, Funding Source ID: U54CA221205
Documents Reviewed:	• JUTH Ethics Review Approval_Cervical , Category: Approval/Authorization Letter;<br>• Consent Form , Category: Consent Form;<br>• Anorlu_CITICertification 2.0, Category: Certificate;<br>• JUTH Ethcial Clearance Approval_2.1, Category: Approval/Authorization Letter;<br>• Musa_CITI Certification , Category: Certificate;<br>• LUTH_RecruitmentFlyer, Category: Recruitment Materials;<br>• Anorlu_CITICertification, Category: Certificate;<br>• Sagay_CITI Certification , Category: Certificate;<br>• Sagay_CITI Certification 2.0, Category: Certificate;<br>• LUTH_Ethical Review Approval_Cervical , Category: Approval/Authorization Letter;<br>• JUTH_RecruitmentFlyer, Category: Recruitment Materials;<br>• LUTH_Ethical Review Approval_2.1, Category: Approval/Authorization Letter;<br>• Ogunsola_CITICertification , Category: Certificate;<br>• Revised Protocol , Category: IRB Protocol;<br>• JUTH IRB RENEWAL FOR U54 PROJECT 2_2022.pdf, Category: Continuing Review Supporting Document;<br>• LUTH IRB Renewal for U54 Project 2 2022.pdf, Category: Continuing Review Supporting Document

Unaffiliated External Site(s) that rely on NU IRB:	• Lagos University Teaching Hospital<br>• Jos University Teaching Hospital

In conducting this study, you are required to follow the requirements listed in the Northwestern University (NU) Investigator Manual (HRP-103), which can be found by navigating to the policy section of the IRB website. Additionally, as Principal Investigator (PI) of this research study, you are expected to adhere to the investigator responsibilities outlined in the "What are my obligations as Investigator in order to conduct Human Research" section of the Investigator Manual (HRP-103).

If you are unable to complete the study within the approval period, you will need to submit a continuing review to renew the study. The continuing review must be submitted no more than 60 days prior to the expiration date.

All Non-Exempt Human Research, including studies without a continuing review, are subject to routine IRB post-approval monitoring as outlined in the "What are my obligations as Investigator in order to conduct Human Research" section of the Investigator Manual (HRP-103) and the "Reporting Concerns" section of the Human Research Protection Program Compliance (HRPP).

NU IRB approval does not constitute or guarantee institutional approval and/or support. Investigators and study team members must comply with all applicable federal, state, and local laws, as well as NU Policies and Procedures, which may include obtaining approval for your research activities from other individuals or entities.

For IRB-related questions, please consult the NU IRB website at http://irb.northwestern.edu. For general research questions, please consult the NU Office for Research website at www.research.northwestern.edu.

COLLEGE OF MEDICINE, UNIVERSITY OF LAGOS
HEALTH RESEARCH ETHICS COMMITTEE
CMUL HREC Registration Number: HREC/15/04/2015
Office Address: 2nd Floor, Biomedical Engineering Block,
College of Medicine, University of Lagos
P.M.B. 12003, Lagos, Nigeria
Telephone: 0802 864 2463 *E-mail:* hrec@cmul.edu.ng *Website:* cmul.unilag.edu.ng

Chairman:
Prof. Sunday A. Omilabu
B.Sc. (Hons.) (Ife), M.Sc. (Ibadan),
Ph.D. (Ibadan)

Vice-Chairman:
Dr. Kolawole S. Oyedeji
B.Sc. (Uniport), M.Sc. (Ife),
MHRE (South Africa), Ph.D, (Lagos),
AIMLS (Nig.), CPHPM (Ilorin)

19th October, 2019

Re: Epigenetic Biomarkers of Cervical Cancer in Nigerian Women

CMULHREC Number: CMUL/HREC/09/19/327
Name of Principal Investigators: Professor Ogunsola, Folasade
Date of receipt of valid application: 9th September, 2019
Date of meeting when final determination of research was made: 16th October, 2019

APPROVAL LETTER FOR AMENDMENT/MODIFICATION

The above named proposal has been adequately reviewed; the protocol and safety guidelines satisfy the conditions of **CMULHREC** policies regarding experiments involving human and or animal participants.

Therefore, the study under its reviewed state is hereby **approved by the Health Research Ethics committee of College of Medicine of the University of Lagos.**

PROF. S.A. OMILABU ———— 5/11/19
Name of CMULHREC Chairman — ***Signature & Date***

Dr. K. S. Oyedeji ———— 5/11/19
Name of CMULHREC Member — ***Signature & Date***

This approval is given with the investigator's responsibility declaration as attached and that;

i) You will submit in CMULHREC prescribed forms, annual progress report during the course of this study, if it is more than one year and final report as the case may be if less than one year and after completion of the study.

ii) The CMULHREC reserves the right to monitor and review this approval; even after the commencement of your study and inform you of any further changes or amendments that may be required for your compliance.

This approval dates from **17/10/2019 to 16/10/2020.** If there is delay in starting the research, please inform the HREC so that the dates of approval can be adjusted accordingly.

COLLEGE OF MEDICINE, UNIVERSITY OF LAGOS
HEALTH RESEARCH ETHICS COMMITTEE

CMUL HREC Registration Number: HREC/15/04/2015

Office Address: 2nd Floor, Biomedical Engineering Block,
College of Medicine, University of Lagos
P.M.B. 12003, Lagos, Nigeria

Telephone: 0802 864 2463 *E-mail:* hrec@cmul.edu.ng *Website:* cmul.unilag.edu.ng

Chairman:
Prof. Sunday A. Omilabu
B.Sc. (Hons.) (Ife), M.Sc. (Ibadan), Ph.D. (Ibadan)

Vice-Chairman:
Dr. Kolawole S. Oyedeji
B.Sc. (Uniport), M.Sc. (Ife), MHRE (South Africa), Ph.D, (Lagos), AIMLS (Nig.), CPHPM (Ilorin)

12th March, 2018

Re: Epigenetic Biomarkers of Cervical Cancer in Nigerian Women

CMULHREC Number: CMUL/HREC/01/18/327

Name of Principal Investigators: Prof F. T. Ogunsola
Date of receipt of valid application: 3rd January, 2018

APPROVAL LETTER

The above named proposal has been adequately reviewed; the protocol and safety guidelines satisfy the conditions of **CMULHREC** policies regarding experiments involving human and or animal participants.

Therefore, the study under its reviewed state is hereby **approved by the Health Research Ethics committee of College of Medicine of the University of Lagos.**

PROF. S.A. OMILABU
Name of CMULHREC Chairman

15/03/18
Signature & Date

Dr. M. O. Akinleye
Name of CMULHREC Member

Akinleye 15/03/2018
Signature & Date

This approval is given with the investigator's responsibility declaration as attached and that;

iii) You will submit in CMULHREC prescribed forms, annual progress report during the course of this study, if it is more than one year and final report as the case may be if less than one year and after completion of the study.

iv) The CMULHREC reserves the right to monitor and review this approval; even after the commencement of your study and inform you of any further changes or amendments that may be required for your compliance.

This approval dates from **12/03/2018 to 11/03/2019.** If there is delay in starting the research, please inform the HREC so that the dates of approval can be adjusted accordingly.

JOS UNIVERSITY TEACHING HOSPITAL
JOS, NIGERIA

Phone: 073-450226-9
E-mail: juth@infoweb.abs.net

Cables & Telegram: JUTH
P.M.B. 2076
JOS

Ref: JUTH/DCS/ADM/127/XXVII/630

Date: 27th November, 2017.

Prof. Solomon A. Sagay,
Department of Obstetrics and Gynaecology,
Jos University Teaching Hospital,
Jos-Nigeria.

RE: ETHICAL CLEARANCE/APPROVAL

I am directed to refer to your application dated 20th November, 2017 on the research proposal titled:

"Epigentic Biomarkers of Cervical Cancer in Nigeria Women" and your appearance before the Ethical Committee on 24th November, 2017.

Following recommendation from the Institutional Health Research Ethics Committee, I am to inform you that Management has given approval for you to proceed on your research topic as indicated.

You are however required to obtain a separate approval for use of patients and facilities from the department(s) you intend to use for your research.

The Principal Investigator is required to send a progress report to the Ethical Committee at the expiration of three (3) months after ethical clearance to enable the Committee carry out its oversight function.

Submission of final research work should be made to the Institutional Health Research Ethical Committee through the **Secretary, Administration Department,** please.

On behalf of the Management of this Hospital, I wish you a successful research outing.

Comfort A. Onoja
For: Chairman, MAC,

INSTITUTIONAL HEALTH RESEARCH ETHICAL COMMITTEE
DESPATCHED
27 NOV 2017

JOS UNIVERSITY TEACHING HOSPITAL
JOS, NIGERIA

Phone: +234 903 0001194
+234 905 8777722
Email: info@juth,org.ng
juthjos@gmail.com

Cables & Telegram: JUTH
P.M.B. 2076
Jos, 930241, NG

JOS UNIVERSITY TEACHING HOSPITAL
HEALTH & SERVICE

Ref: JUTH/DCS/ADM/127/XIX/5962

Date: 9th February, 2022

Dr. Solomon A. Sagay,
Department of Obstetrics and Gynaecology,
Jos University Teaching Hospital,
Jos, Plateau State.

RE: APPLICATION FOR RENEWAL (RE-VALIDATION) OF ETHICAL CLEARANCE

I write to refer to your letter on the above subject dated 2nd February, 2022, and to inform you that Management has approved the renewal (re-validation) of ethical clearance in respect of the research topic title:

"**Epigenomic Biomarkers of Cervical Cancer in Nigeria Women** with effect from 9th February, 2022 to 9th February, 2023.

However, on completion of your research work, it is mandatory that you submit two (2) copies of the report to Administrative Secretary, of Ethical Committee in Room 14, Administration Department, JUTH, please.

On behalf of Management of this Hospital, I wish you a successful research outing.

LOHDIR NANA NIMAC
For: Chairman MAC

RESEARCH & ETHICS COMMITTEE
09 FEB 2022
JOS UNIVERSITY TEACHING HOSPITAL

COLLEGE OF MEDICINE, UNIVERSITY OF LAGOS

HEALTH RESEARCH ETHICS COMMITTEE

CMUL HREC Registration Number: NHREC/19/08/2019B

Office Address: 2nd Floor Biomedical Engineering Block
College of Medicine University of Lagos, Idi-Araba, Lagos
P.M.B.12003, Lagos Nigeria
Telephone: 08028642463, 08025187265, 08023119431
E-mail: hrec@cmul.edu.ng ***website***: *cmul.unilag.edu.ng*

Chairman:
Prof. Sunday A. Omilabu
BSc. (Hons), Ife, MSc. (Ibadan)
PhD. (Ibadan)

Vice Chairman:
Prof. Kolawole S. Oyedeji
BSc (Uniport) MSc (Ife), MSc (Unilag)
MSocSci-HRE (South Africa), FMLSCN (Nig)
CPHPM (Ilorin), PhD (Unilag).

4th December, 2020

Re: Epigenetic Biomarkers of Cervical Cancer in Nigerian Women

CMULHREC Number: CMUL/HREC/09/19/327
Name of Principal Investigators: Prof. Folasade Ogunsola
Date of receipt of valid application: 23rd November, 2020
Date of meeting when final determination of research was made: 4th December, 2020

APPROVAL LETTER FOR AMENDMENT

The above named proposal has been adequately reviewed; the protocol and safety guidelines satisfy the conditions of **CMULHREC** policies regarding experiments involving human and or animal participants.

Therefore, the study under its reviewed state is hereby **approved by the Health Research Ethics committee of College of Medicine of the University of Lagos.**

PROF. S.A. OMILABU ------------------------------------07/12/2020
Name of CMULHREC Chairman ***Signature & Date***

Dr. K. S. Oyedeji ----- ------------------------------
Name of CMULHREC Member ***Signature & Date 09/12/2020***

This approval is given with the investigator's responsibility declaration as attached and that;

i) You will submit in CMULHREC prescribed forms, annual progress report during the course of this study, if it is more than one year and final report as the case may be if less than one year and after completion of the study.

ii) The CMULHREC reserves the right to monitor and review this approval; even after the commencement of your study and inform you of any further changes or amendments that may be required for your compliance.

This approval dates from **04/12/2020 to 03/12/2021.** If there is delay in starting the research, please inform the HREC so that the dates of approval can be adjusted accordingly.

COLLEGE OF MEDICINE, UNIVERSITY OF LAGOS

HEALTH RESEARCH ETHICS COMMITTEE

CMUL HREC Registration Number: NHREC/19/08/2019B

Office Address: 2nd Floor Biomedical Engineering Block
College of Medicine University of Lagos, Idi-Araba, Lagos
P.M.B.12003, Lagos Nigeria
***Telephone*:** 08028642463, 08025187265, 08023119431
***E-mail*:** hrec@cmul.edu.ng ***website*:** *cmul.unilag.edu.ng*

Chairman:
Prof. Kolawole S. Oyedeji
BSc (Uniport) MSc (Ife), MSc (Unilag)
MSocSci-HRE (South Africa), FMLSCN (Nig)
CPHPM (Ilorin), PhD (Unilag).

Vice Chairman:
Dr. Oluwakemi O. Odukoya
MBBS (Lagos), MPH (Lagos), M.Sc.
MWACP, FMCPH.

17th March, 2022
Re: Epigenetic Biomarkers of Cervical Cancer in Nigerian Women

CMULHREC Number: CMUL/HREC/02/22/327/V4
Name of Principal Investigators: Professor Ogunsola, Folasade
Date of receipt of valid application: 05th February, 2022
Date of meeting when final determination of research was made: 17th March, 2022

ANNUAL RE: APPROVAL LETTER

The above named proposal has been adequately reviewed; the protocol and safety guidelines satisfy the conditions of CMULHREC policies regarding experiments involving human and or animal participants.

Therefore, the study under its reviewed state is hereby **approved by the Health Research Ethics committee of College of Medicine of the University of Lagos.**

Prof. Kolawole S. Oyedeji 19-03-2022
Name of CMULHREC Chairman ***Signature & Date***

Dr. Oluwakemi O. Odukoya 18/03/2022
Name of CMULHREC Member ***Signature & Date***

This approval is given with the investigator's responsibility declaration as attached and that;

i) You will submit in CMULHREC prescribed forms, annual progress report during the course of this study, if it is more than one year and final report as the case may be if less than one year and after completion of the study.

ii) The CMULHREC reserves the right to monitor and review this approval; even after the commencement of your study and inform you of any further changes or amendments that may be required for your compliance.

This approval dates from **17/03/2022 to 16/03/2023.** If there is delay in starting the research, please inform the HREC so that the dates of approval can be adjusted.

Northwestern | RESEARCH

Northwestern University
Institutional Review Board
Biomedical IRB
750 N. Lake Shore Dr., 7th Fl.
Chicago, Illinois 60611

irb@northwestern.edu
Office 312. 503. 9338

Social & Behavorial Sciences IRB
600 Foster St., 2nd Floor
Evanston, Illinois 60208

sbsirb@northwestern.edu
Office 847. 467. 1723

APPROVAL OF NEW STUDY

DATE: June 1, 2018

TO: Lifang Hou
FROM: Office of the IRB

DETERMINATION DATE: 6/1/2018
APPROVAL DATE: 5/24/2018
EXPIRATION DATE: 5/23/2019

The Northwestern University IRB reviewed and approved the submission described below:

Type of Submission:	Initial Study
Review Level:	Committee
Title of Study:	Epigenetic biomarkers of cervical cancer in Nigerian Women
Principal Investigator:	Lifang Hou
IRB ID:	STU00207051
Funding Source:	Name: National Cancer Institute, Grant Office ID: SP0041943, Funding Source ID: U54CA221205
Grant ID:	SP0041943;
IND, IDE, or HDE:	None
Documents Reviewed:	• JUTH Ethics Review Approval_Cervical , Category: Approval/Authorization Letter;<br>• Consent Form , Category: Consent Form;<br>• Musa_CITI Certification , Category: Certificate;<br>• LUTH_RecruitmentFlyer, Category: Recruitment Materials;<br>• Anorlu_CITICertification, Category: Certificate;<br>• NU_eIRB_Cervical_Cancer project_2018_Feb9 revised 21Feb2018.docx, Category: IRB Protocol;<br>• Full Grant , Category: Sponsor Attachment;<br>• Sagay_CITI Certification , Category: Certificate;<br>• LUTH_Ethical Review Approval_Cervical , Category: Approval/Authorization Letter;<br>• JUTH_RecruitmentFlyer, Category: Recruitment Materials;<br>• Protocol , Category: IRB Protocol;<br>• Ogunsola_CITICertification , Category: Certificate;
Special Determination(s):	Study constitutes greater than minimal risk and NU holds the federal grant.

In conducting this study, you are required to follow the requirements listed in the Northwestern University (NU) Investigator Manual (HRP-103), which can be found by navigating to the IRB Library within the eIRB+ system.

If you are unable to complete the study within the approval period, you will need to submit a continuing review to renew the study. The continuing review must be submitted no more than 60 days and no less than 30 days prior to the expiration date.

NU IRB approval does not constitute or guarantee institutional approval and/or support. Investigators and study team members must comply with all applicable federal, state, and local laws, as well as NU Policies and Procedures, which may include obtaining approval for your research activities from other individuals or entities.

For IRB-related questions, please consult the NU IRB website at http://irb.northwestern.edu. For general research questions, please consult the NU Office for Research website at www.research.northwestern.edu.

JOS UNIVERSITY TEACHING HOSPITAL
JOS, NIGERIA

Phone: +234 903 0001194
+234 905 8777722
Email: info@juth.org.ng
juthjos@gmail.com

HEALTH AND SERVICE

Cables & Telegram: JUTH
P.M.B. 2076
Jos, 930241, NG

Ref: JUTH/DCS/ADM/127/XXVII/630

Date: 3rd September, 2019

Prof. Solomon A. Sagay,
Department of Obstetrics and Gynaecology,
Jos University Teaching Hospital,
Plateau State-Nigeria.

RE: APPROVALOF PROTOCOL AMENDMENT

I am directed to refer to your application dated 22nd August, 2019 on the research proposal titled:

"Epigentic Biomarkers of Cervical Cancer in Nigerian Women"

Following recommendation from the Institutional Research Ethical Committee, I am to inform you that Management has given approval for you to proceed on your research topic as indicated.

1. We have modified the protocol and informed consent for additional sample collections (cervical swabs and cervico-vaginal lavage specimens).
2. Modification of aim 3 to include HIV positive women with CIN 2 and 3 for enrollment (details provided in the protocol).
3. Inclusion of relevant statements on "material transfer agreement" permitting shipment and sharing of shipped specimens to other collaborating institutions in US (Mayo clinic inclusive).

On behalf of the Management of this Hospital, I wish you a successful research outing.

Julcit E. Vyapbong
For: Chairman, MAC,

INSTITUTIONAL HEALTH RESEARCH ETHICS COMMITTEE
[illegible]
03 SEP 2019
JOS UNIVERSITY TEACHING HOSPITAL
P.M.B. 2076, JOS - NIGERIA
