# Supplementary material for: Epidemiology and survival outcomes of HIV-associated cervical cancer in Nigeria
Source: Infect Agent Cancer. 2023 Nov 1;18:68. doi: 10.1186/s13027-023-00550-7 (PMC10619301; doi:10.1186/s13027-023-00550-7)
Supplement: Supplementary file 2 — Additional file 2: Scanned histopathologic images of cervical cancer with tumor grading [file 13027_2023_550_MOESM2_ESM.docx]

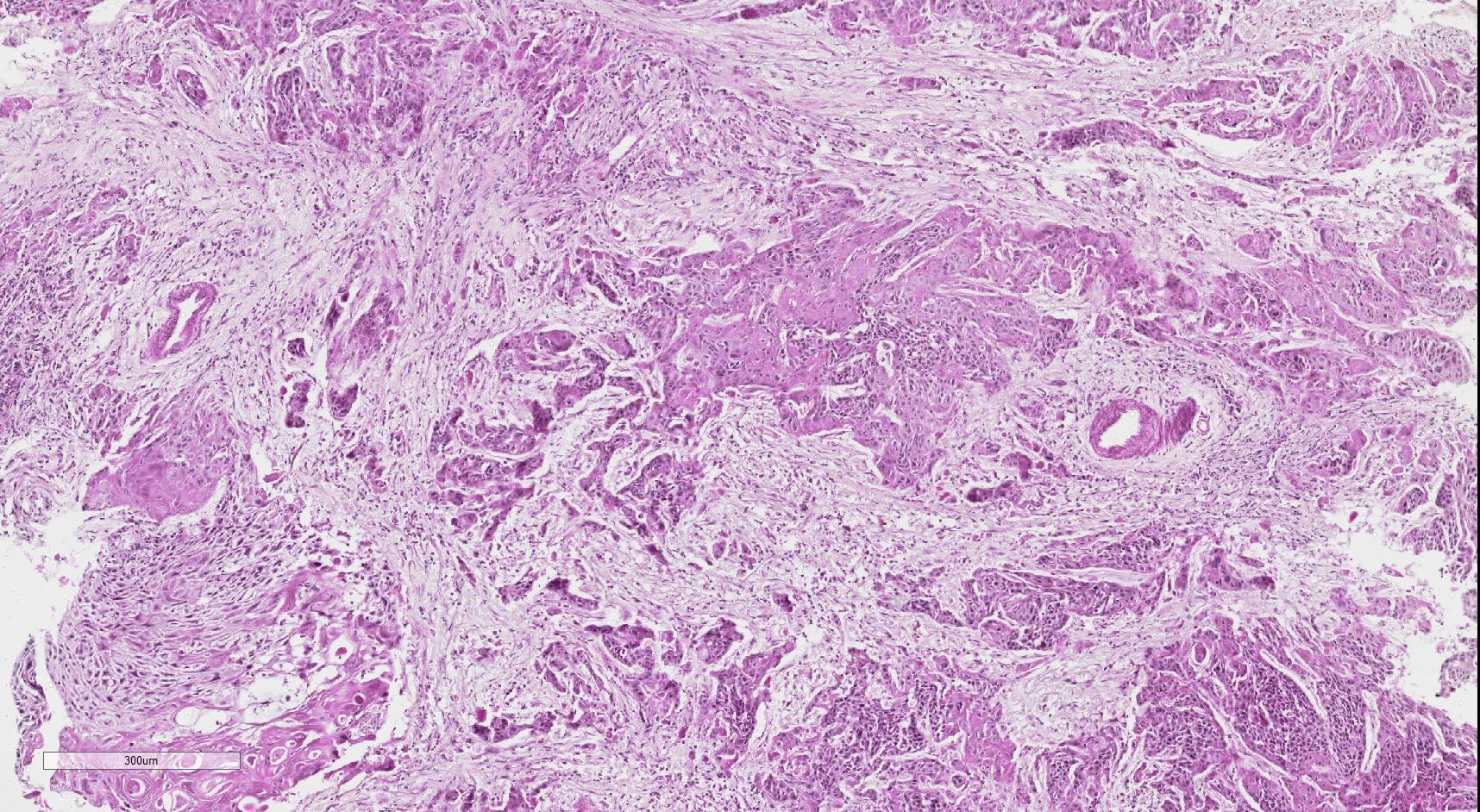


Moderate differentiated squamous cell carcinoma


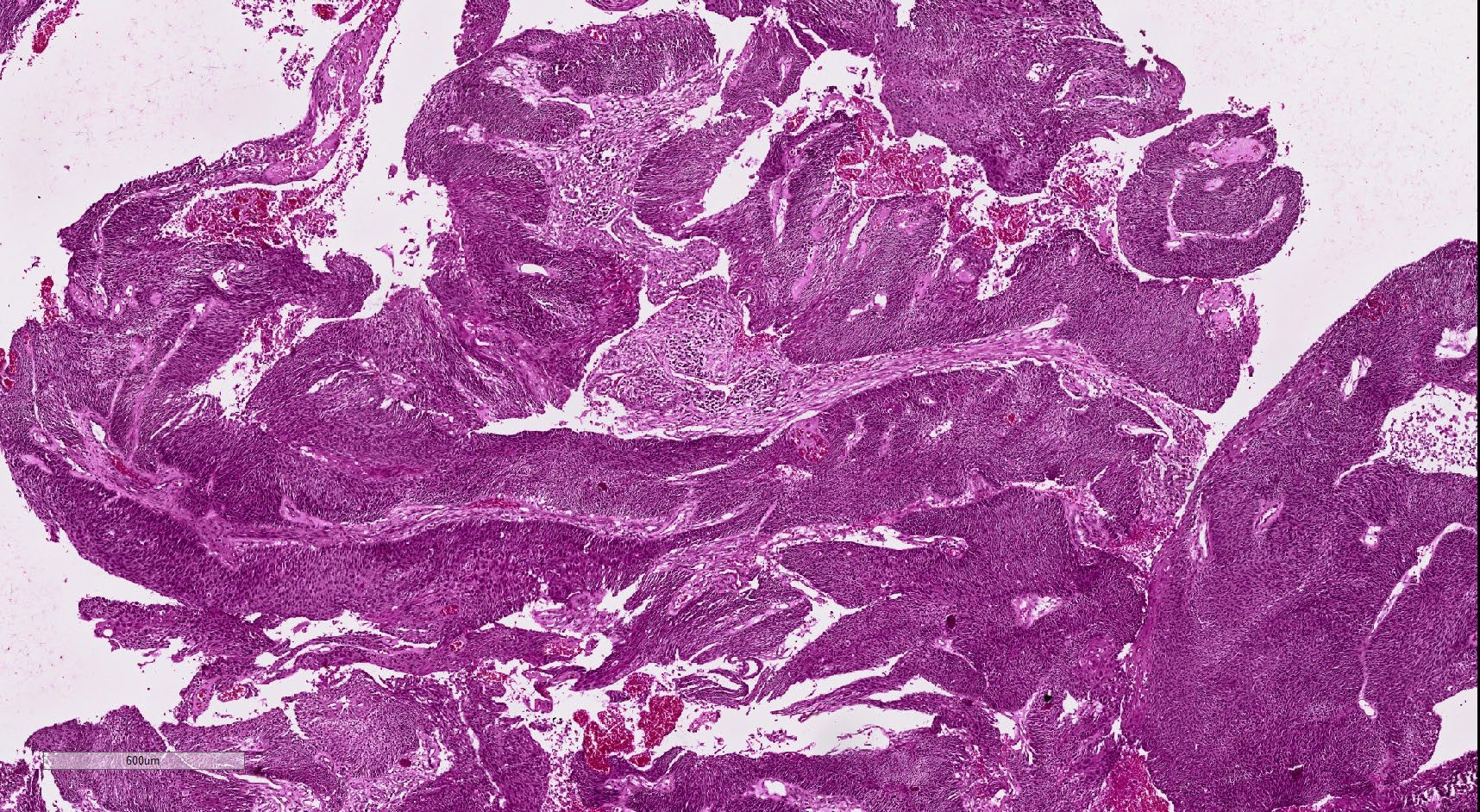


Papillary squamous cell carcinoma, basaloid type


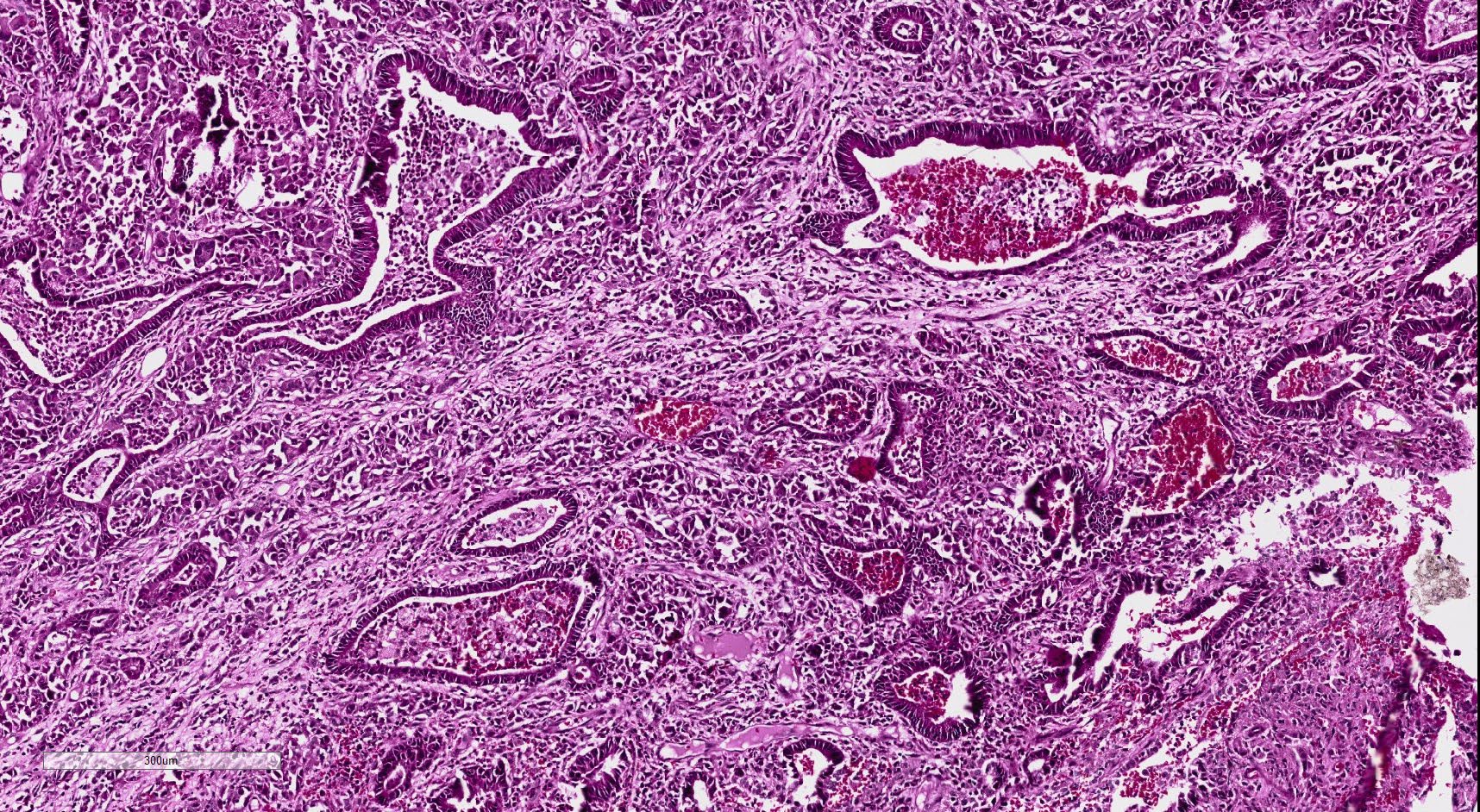


Cervical adenocarcinoma


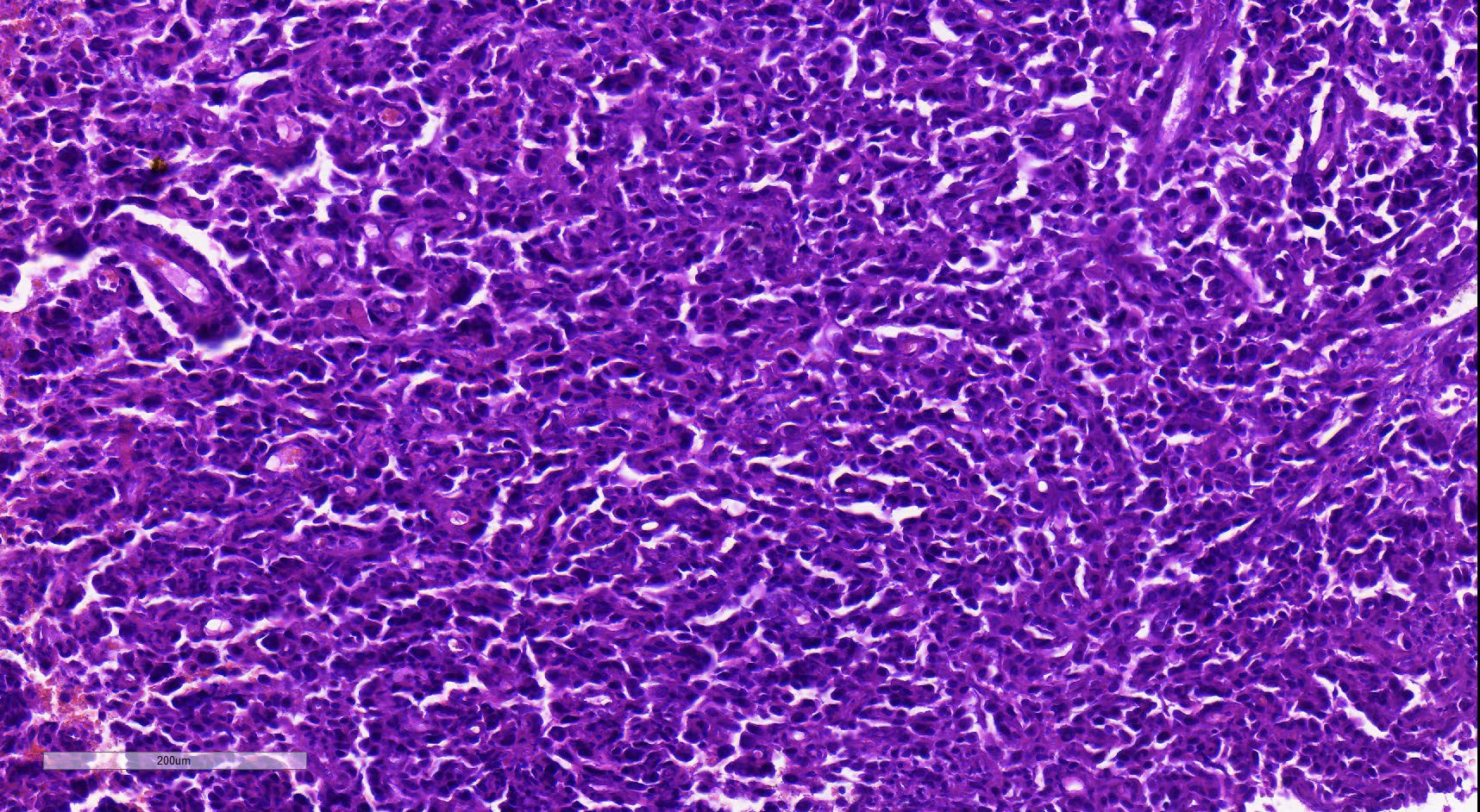


Poorly differentiated squamous cell carcinoma


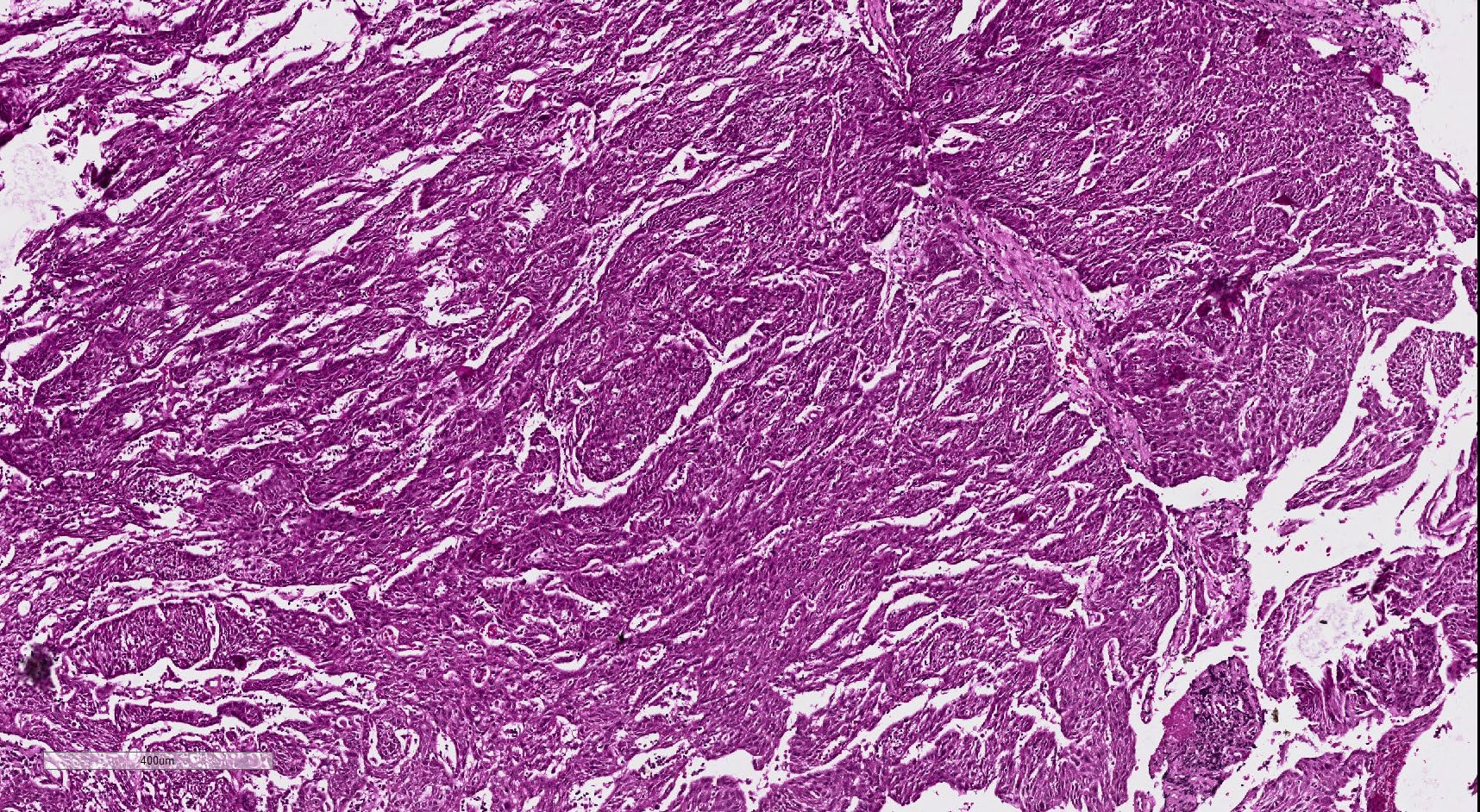


Poorly differentiated squamous cell carcinoma


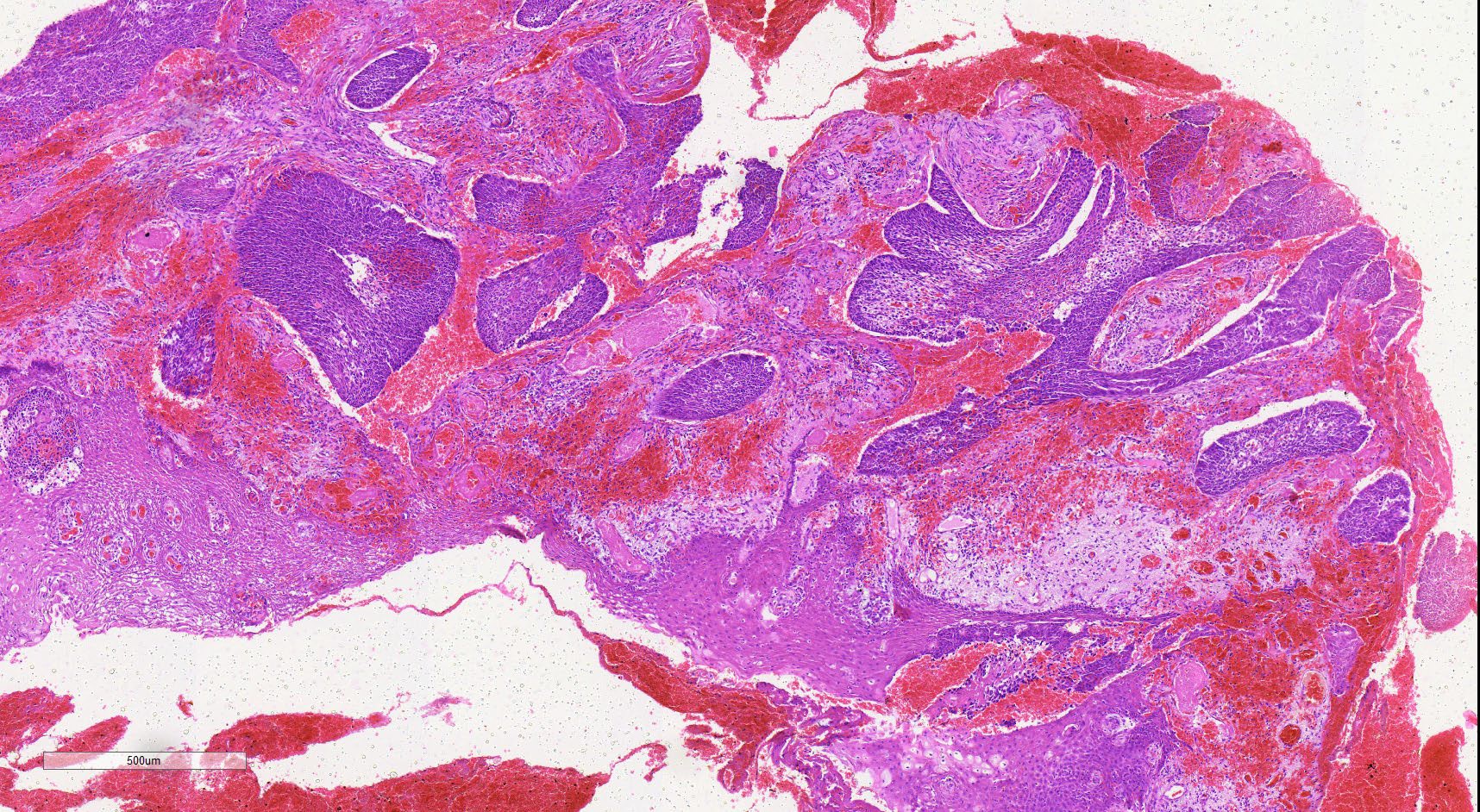


Well differentiated squamous cell carcinoma
